# Biodegradable Polyphosphazene Biomaterials for Tissue Engineering and Delivery of Therapeutics

**DOI:** 10.1155/2014/761373

**Published:** 2014-04-29

**Authors:** Amanda L. Baillargeon, Kibret Mequanint

**Affiliations:** ^1^Graduate Program of Biomedical Engineering, Faculty of Engineering, The University of Western Ontario, London, ON, Canada N6A 5B9; ^2^Department of Chemical & Biochemical Engineering, Faculty of Engineering, The University of Western Ontario, London, ON, Canada N6A 5B9

## Abstract

Degradable biomaterials continue to play a major role in tissue engineering and regenerative medicine as well as for delivering therapeutic agents. Although the chemistry of polyphosphazenes has been studied extensively, a systematic review of their applications for a wide range of biomedical applications is lacking. Polyphosphazenes are synthesized through a relatively well-known two-step reaction scheme which involves the substitution of the initial linear precursor with a wide range of nucleophiles. The ease of substitution has led to the development of a broad class of materials that have been studied for numerous biomedical applications including as scaffold materials for tissue engineering and regenerative medicine. The objective of this review is to discuss the suitability of poly(amino acid ester)phosphazene biomaterials in regard to their unique stimuli responsive properties, tunable degradation rates and mechanical properties, as well as *in vitro* and *in vivo* biocompatibility. The application of these materials in areas such as tissue engineering and drug delivery is discussed systematically. Lastly, the utility of polyphosphazenes is further extended as they are being employed in blend materials for new applications and as another method of tailoring material properties.

## 1. Introduction

Over the past few decades, tissue engineering and regenerative medicine have become significant areas of research due to their potential to fix or replace damaged tissues and prolong life [1, 2]. Tissue engineering and regenerative medicine incorporate knowledge from the areas of biology, materials science, and engineering to repair, restore, and regenerate living tissues that may have been compromised by disease, injury, or other means [3, 4]. Combining the expertise from these disciplines along with the development and application of biomaterials, cells, and bioactive molecules such as growth factors, tissue-engineered products, and regenerative medicine strategies that are capable of extending lifespans and overcoming numerous health problems is made possible [3, 5, 6]. Not surprisingly, the development of suitable biomaterials, including a variety of polymers and ceramics, which are critical for the success of tissue engineering and regenerative medicine, is being explored [7, 8]. Depending on the target tissue to be engineered, the biomaterial that is used must exhibit several key characteristics, such as biocompatibility, biostability, or biodegradability, and suitable mechanical properties (e.g., tensile strength and compression resistance).

Biomaterials for tissue engineering must be biocompatible since they eventually must be implanted into the patient and a prolonged immune response would be problematic [9]. Natural polymers such as chitosan, collagen, and gelatin are known to be highly biocompatible and therefore have been extensively studied as biomaterials for tissue engineering and other biomedical applications [4, 10]. Their main drawbacks are their inadequate mechanical strength, uncontrolled degradation rates, and poorly defined structure [10, 11]. This has lead researchers to investigate synthetic polymers as an alternative to natural materials. Biodegradability is a desirable feature of a biomaterial used in tissue engineering since the goal is that it acts as a temporary scaffold holding the growing tissue in place until the natural extracellular matrix has sufficiently developed. Beyond that point, the scaffold should breakdown into nontoxic degradation products capable of being disposed of by the body leaving only the newly formed tissue. There are a wide variety of synthetic biodegradable polymers that have been, and continue to be, explored including polyesters, polyanhydrides, polyacetals, and poly(*α*-amino acids) [10]. Despite their improvement over natural polymers with regard to degradation and mechanical properties, synthetic polymers have their own limitations. A common problem of synthetic polymers such as poly(lactic acid) (PLA) is the formation of acidic products during the degradation process which leads to diminished mechanical strength of the material and compromised cell function in the acidic environment [12–14]. The quest for biomaterials with tunable degradation rates and mechanical properties, which also maintain cell function and lack the formation of toxic degradation products, is an active area of research [15, 16].

**Figure 1:**
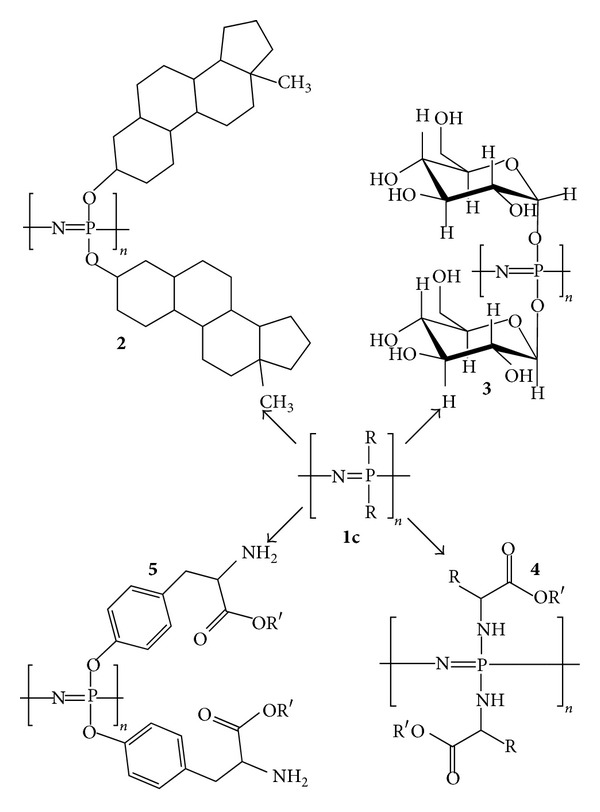
Structures of various polyphosphazenes including steroidal substituents (**2**), carbohydrates (**3**), amino acid esters (**4**), and side chain-bound amino acid esters (**5**), to name a few. Adapted from [5] by permission of the Royal Society of Chemistry (http://dx.doi.org/10.1039/B926402G).

Sustained research towards new biomaterials for tissue engineering and regenerative medicine applications has led to the utilization of polyphosphazenes as a class of novel materials. Polyphosphazenes are comprised of an inorganic backbone of repeating phosphorus and nitrogen atoms with alternating single and double bonds (Figure 1, Structure** 1c**) [6, 17–19]. Extending from each of the phosphorus atoms are two organic side chains, which can range from alkoxy and aryloxy substituents to amino acids, giving a large variety of potential polymers [5, 18, 20, 21]. Changing the organic side groups and their ratios, if multiple different side groups are attached to the same polymer backbone, allows substantial tunability of the physical and degradation properties of the material [18, 22, 23]. Therefore, altering the organic substituents can be quite useful in tailoring the mechanical properties and degradation rates of the biomaterial to suit the desired tissue engineering application, such as bone tissue or blood vessels, which require drastically different physical properties [24, 25].

## 2. Synthesis of Polyphosphazenes

The synthesis of polyphosphazenes such as those shown in Figure 1 is typically via a two-step reaction beginning with the thermal ring opening polymerization of hexachlorocyclotriphosphazene (**1a**), the cyclic trimer, to the linear poly(dichlorophosphazene) (**1b**) precursor. Next, the organic side chains are attached to the polymer backbone through a nucleophilic macromolecular substitution of the organic substituents for the phosphorus-bound chlorine atoms [18, 21, 26]. The following two sections succinctly will describe the individual steps of polyorganophosphazene synthesis showing the vast range of materials that can be generated.

### 2.1. Thermal Ring Opening Polymerization-Bulk Phase

**Scheme 1:**
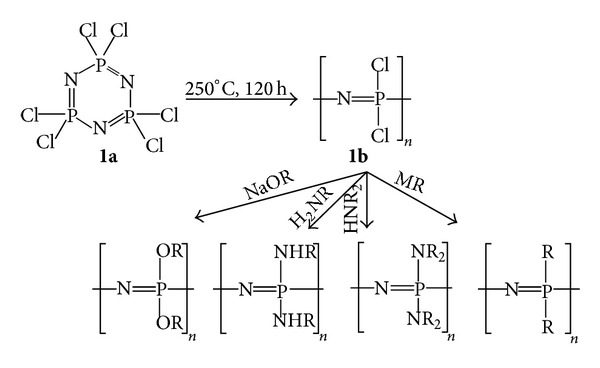
Scheme showing the synthesis and functionalization of poly(dichlorophosphazene) (**1b**) in the overall synthesis of polyphosphazenes from hexachlorocyclotriphosphazene (**1a**). Reproduced from [5] by permission of the Royal Society of Chemistry (http://dx.doi.org/10.1039/B926402G).

Although the thermal ring polymerization of the trimer (**1a**) to linear poly(dichlorophosphazene) was attempted in the late 1800s by H. N. Stokes, a useful material that was soluble and capable of being functionalized was not realized until the 1960s. The initial thermal ring opening polymerization performed by Stokes lead to a product that was insoluble, due to crosslinking, and that was readily susceptible to hydrolysis when exposed to moisture [18]. In 1965, Allcock and Kugel [27] were able to synthesis linear poly(dichlorophosphazene) through a well-controlled thermal ring opening polymerization from the cyclic trimer hexachlorocyclotriphosphazene according to Scheme 1. The product obtained was soluble allowing it to be modified further by macromolecular substitution of the reactive P–Cl bonds with organic and organometallic nucleophiles. The thermal ring opening polymerization technique developed by Allcock et al. is the most commonly used route to prepare the linear poly(dichlorophosphazene) precursor [5, 18]. A typical process involves reacting purified hexachlorocyclotriphosphazene trimer at 250°C over 5 days in an evacuated polymerization tube. At this point, soluble poly(dichlorophosphazene) has been formed that can be purified and functionalized via the macromolecular substitution reaction (Scheme 1,** 1a→1b**) [18]. Despite the success of the bulk phase thermal ring opening polymerization in lab scale syntheses, this method is not economically feasible for large-scale production of polyphosphazene materials. Alternative methods, which will not be discussed in this review, including solution phase thermal ring opening [28], living cationic [29–32], and one-pot De Jaeger [33] polymerization techniques have been reported.

### 2.2. Functionalization of the Poly(dichlorophosphazene) Precursor

Once high molecular weight linear poly(dichlorophosphazene) is synthesized, the polymer can be modified by substituting the phosphorus-bound chlorine atoms with organic side groups. The polymer undergoes a macromolecular substitution (Scheme 1, reaction of compound** 1b** into four potential polyphosphazene structures) when subjected to organic and organometallic nucleophiles forming a large class of polyphosphazenes as shown in Figure 1 [18, 26]. All of the polyphosphazenes shown in Figure 1 have one type of side chain throughout the entire polymer, although it has been shown that cosubstituted materials with well-defined ratios of side chains are possible by controlling the amount and order addition of the nucleophiles [18, 21, 34]. The modification of type and ratios of the side chains of the polymer affords the ability to fine-tune degradation rates and physical properties based on these substituents, which is important to the synthesis of a biomaterial suitable for tissue engineering and therapeutic delivery [23].

## 3. Suitability of Polyphosphazene Biomaterials

**Figure 2:**
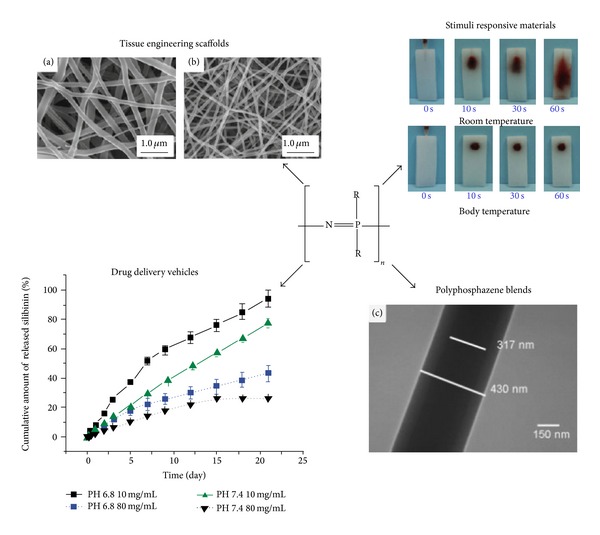
Overview of several biomedical applications where polyphosphazenes have been shown to be useful biomaterials Reprinted from [50–53] with permission from Springer Science, Business Media, and Elsevier.

In order for polyphosphazenes to be considered a suitable biomaterial, they must be compatible with the biological environment they are intended to interact with [5, 35, 36]. They must also be either biostable or biodegradable into nontoxic degradation products. Bioerodible biomaterials are usually preferred since they leave only the natural tissue once the material has degraded, eliminating the long-term risk of immune response and potential negative outcome [10, 23, 35, 37–47]. Lastly, the biomaterial must have mechanical properties that match or closely resemble those of the natural tissue so that issues such as compliance mismatch, a common problem, for example, in vascular tissue engineering, are reduced [36, 48]. In the next few sections, we summarize the current understanding regarding the biocompatibility, biodegradation, and mechanical properties of polyphosphazene biomaterials. The remainder of this review will focus on the suitability of polyphosphazenes, mainly poly(amino acid ester)phosphazenes, as biomaterials due to their unique tunability of degradation and mechanical properties making them useful in a wide range of biomedical applications as is shown in Figure 2 [5, 21, 49].

### 3.1. Stimuli Responsive Polyphosphazenes

Wilfert et al. [54] manipulated the biodegradability of polyphosphazenes and were capable of developing materials with well-controlled pH responsive degradation rates that were also water soluble. They synthesized materials with side chains including poly(ethylene oxide-copropylene oxide) (M-1000) alone, valine spaced M-1000, and glycine spaced M-1000. Degradation studies were performed by placing 20 mg samples of the materials in deuterated water (D_2_O) of varying pH (2, 5, and 7.6) and monitoring changes in GPC traces, ^31^P nuclear magnetic resonance (NMR) spectra, and ultraviolet-visible (UV-Vis) spectroscopy spectra. It was shown that the material without any amino acid linkers degraded much more slowly than those with valine or glycine linkers between the M-1000 and the polyphosphazene backbone. It was also demonstrated that the polymers degraded more quickly in the presence of acid with degradation rates of polyphosphazenes in pH 2 being fully degraded in the time span of days, whereas polymers in neutral pH conditions (pH = 7.6) degraded much more slowly on the order of months and a substantial amount of the starting polymer remained after the 4 week study period. In order for these materials to be useful as drug carriers with stimuli responsive degradation properties they must also be biocompatible and it was shown that their degradation products did not significantly impact cell viability. This study shows strong support of pH responsive and water-soluble polyphosphazene-based materials for their use in drug delivery applications.

Thermoresponsive degradable polyphosphazenes containing lactic acid ester and methoxyethoxy ethoxy side chains for use in biomedical applications were investigated by Bi and coworkers [55]. Three polymers were synthesized with different lengths of lactic acid ester alkyl chains ranging from ethyl to butyl. The polymers with the butyl lactic acid ester had decreased lower critical solution temperatures (LCST) in comparison to those with ethyl esters indicating that they change from a solution to a precipitate gel at lower temperatures. This is due to the fact that the butyl chains are more hydrophobic than the ethyl esters causing the materials to experience more hydrophobic interactions at lower temperatures. Increased hydrophobic interactions lead to the exclusion of water from the polymer and the transition of the polymer from a solution to a gel. The LCSTs of all three materials were between 33°C and 52°C making them useful in biological applications such as drug delivery. Since these materials were being considered for* in vivo *applications their degradation characteristics and biocompatibility were also tested. MTT studies showed that the materials themselves and their degradation products were nontoxic to HepG2 and K562/VCR cells. The biocompatibility and capability to tune the thermoresponsive properties of these polyphosphazene materials indicate their utility as materials for biomedical applications such as drug delivery, especially if localized injection is critical to the treatment plan.

### 3.2. *In Vitro* and* In Vivo* Compatibility of Polyphosphazenes

The cytocompatibility of amino acid ester functionalized polyphosphazene biomaterials was first studied by Laurencin et al. [43] who compared rat primary osteoblast adhesion to poly[(ethyl glycinato) phosphazene] (PNEG) with well-known poly(lactic acid-co-glycolic acid) (PLAGA) and poly(anhydrides). Data from this study showed that the osteoblast cells adhered to the PNEG material to the same extent as the control materials for a period of 8 hours. The degradation of PNEG did not influence cell proliferation as it promoted cell growth to the same extent as the PLAGA control material. In a follow-up study [56], similar experiments on other ethyl glycinato/methyl phenoxy cosubstituted polyphosphazenes using MC3T3-E1 cells (osteoblast precursor cell line from mice) were conducted. The results from this study also suggested that cells responded favourably to polyphosphazene materials, especially those with a high ratio of ethyl glycinato substituents and that cell adhesion and proliferation characteristics were not diminished in comparison to tissue culture plate and PLAGA controls. The polymers with 50% and greater of ethyl glycinato substituents demonstrated improved cell growth in comparison to the tissue culture plate and the polymer with 25% ethyl glycinato substitution was only slightly less effective than the tissue culture plate, although all of these were better than the PLAGA control, which has been widely accepted as a biocompatible material. Studies on cosubstituted amino acid ester-based polyphosphazenes containing an ethyl alanato substituent along with aryloxy substituents such as poly[(ethyl alanato)_1_(ethyl oxybenzoate)_1_ phosphazene] (PNEAEOB) and poly[(ethyl alanato)_1_(propyl oxybenzoate)_1_phosphazene] (PNEAPOB) demonstrated that neither PNEAEOB nor PNEAPOB posed a threat to cell growth, in comparison to the controls, as both materials were capable of promoting cell adhesion and proliferation [47]. Collectively, the results of the above studies from the Laurencin laboratory are promising since cell adhesion and proliferation are not affected in comparison to materials that have previously been extensively studied for their effect on cell viability. One possible drawback with these studies, however, is the cell sources (rat and mouse) that may not appropriately represent what would occur with primary human cells since cell interactions with the materials may not be identical across species. A more suitable cell type to use would be human osteoblasts to get a better indication of how the cells might react to the biomaterial* in vivo* with human subjects.

Gümüşderelioğlu and Gür [57] performed a study that investigated the cytotoxicity of poly[bis(ethyl-4-aminobutyro)phosphazene] by analyzing the activity level of succinic dehydrogenase (SDH) through an MTT assay method. SDH plays a critical role in cellular metabolism and is therefore a good indicator of cytotoxicity [58]. For these experiments, extracts collected from the incubation of the polymeric films with growth medium were added to 3T3 and HepG2 cells. For the negative control, extracts were collected from a polyethylene centrifuge tube that was incubated with the growth medium but lacked a sample of polymeric film. It was shown that poly[bis(ethyl-4-aminobutyro)phosphazene] extracts did not significantly decrease cell viability in Swiss 3T3 and HepG2 cells in comparison to negative controls. The material maintained cell viability, as demonstrated by SDH activity level, greater than 80% of that of the control for all time points and for both cell types. This study was successful in showing the cytocompatibility of the material with respect to 3T3 and HepG2 cells, which are commonly used cell lines to study fibroblast and hepatocyte biology, respectively. The fact that the cells studied are cell lines rather than primary cells is concerning since cell lines are known to grow well, even when conditions may not be ideal. As such, they may not properly represent how the natural tissues, which are not composed of cell lines but rather of primary cells, would respond to the material. Also, it should be noted that the 3T3 cells come from a Swiss mouse source and therefore, just as with the research performed by Laurencin et al., the results may not be indicative of how human cells would react to the material. The cytocompatibility of electrospun matrices of cosubstituted poly(amino acid ester)phosphazenes towards rat endothelial cells was investigated by Carampin and coworkers [59]. They studied poly[(ethyl phenylalanato)_1.4_(ethyl glycinato)_0.6_phosphazene] for both cell adhesion and growth properties in comparison to a fibronectin coated polystyrene tissue culture plate as the control. They found that the polymer only slightly improved cell adhesion (7% increase) in comparison to the culture plates but that the polymer enhanced growth of the adhered cells by approximately 17%. These results reinforced the notion that polyphosphazenes could act as a biocompatible material for use in biomedical applications such as tissue engineering. Again, these results must be considered with caution as they did not use human cells for their research. However, they did use primary cells, which are more sensitive to their environment than cell lines and are an improvement over cell line-based studies.

All of the aforementioned studies involved only* in vitro* analyses of the cytocompatibility of the polyphosphazenes despite the fact that their end goal is to be used as a biomaterial* in vivo*. Towards this end,* in vivo* studies of alanine-modified polyphosphazenes on rat and rabbit models for bone tissue engineering materials have been reported [5, 47]. In the rat model [47], subcutaneously implanted samples were monitored for biocompatibility through immune response. Inflammatory responses were categorized as minimal, mild, or moderate based on the accumulation of immune response cells (e.g., neutrophils/PMNs and lymphocytes) at the implantation site. It was observed that, at 2 weeks after implantation, [poly(ethyl alanato) phosphazene] (PNEA) induced a moderate inflammatory response that initially decreased to minimal at 4 weeks but then slightly increased to mild at 12 weeks. As for poly[(ethyl alanato)_1_(p-methyl phenoxy)_1_ phosphazene] (PNEAmPh), the material caused a moderate inflammatory response at 2 weeks, which gradually decreased to a minimal response after 12 weeks. The poly[(ethyl alanato)_1_ (p-phenyl phenoxy)_1_ phosphazene] (PNEAPhPh) material elicited a mild initial response at 2 weeks, which slowly decreased to a minimal response at 12 weeks. Overall, the inflammatory responses for the PNEAmPh and PNEAPhPh were minimal suggesting that the materials are suitable for bone tissue engineering. The PNEA material triggered a greater inflammatory response than the two cosubstituted polyphosphazenes although the response decreased over the time span of the study suggesting that it is a good candidate, too. All three materials elicited immune responses that were acute and did not pose a long-term threat to the animals.

The potential utility of polyphosphazenes is not limited to bone tissue engineering. Langone et al. [60] conducted* in vivo* biocompatibility of polyphosphazenes as tubular nerve guides in rat models. Comparative studies of poly[(ethyl alanato)_1.4_(imidazolyl)_0.6_phosphazene] (PNEAIL) nerve guides with traditional biostable silicone guides suggested the absence of inflammatory response to the polyphosphazene material after 30 and 60 days of implantation. Upon removal of the implanted nerve guide, it was noted that the stumps of the rat sciatic nerve, which had initially been transected, had reattached with components comprised primarily of nerve fibre bundles, akin the natural nerve tissue. This* in vivo* study suggested that PNEAIL was a biocompatible material, especially for use in nerve regeneration strategies, and highlighted its potential utility in the future. Like the work of Laurencin's group [47], the materials were analyzed in small animal models, which do not behave identically to humans and therefore can only be used as a guideline towards how the materials might respond in a clinical sense.

### 3.3. Biodegradability of Polyphosphazenes

**Scheme 2:**
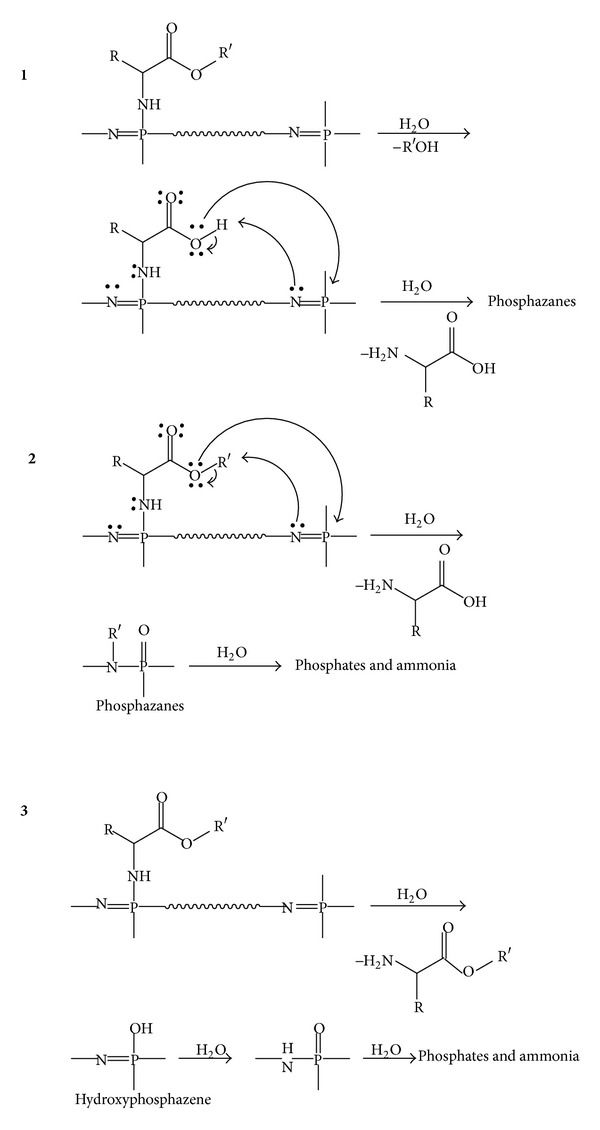
Proposed degradation pathways for the hydrolysis of poly(amino acid ester)phosphazenes. Reprinted with permission from [37]. Copyright 1994 American Chemical Society.

Since it is desirable to use biodegradable biomaterials for tissue engineering therapeutic delivery, many research groups have studied the degradation properties of polyphosphazenes [22, 37–40, 42, 44–47]. Polyphosphazenes are attractive because they have been shown to degrade into nontoxic byproducts that are easily metabolized by the body. In the case of an amino acid ester phosphazenes, these hydrolytic degradation products include the amino acid, the corresponding alcohol of the ester, ammonia, and phosphates [37]. Unlike the acidic products produced from the hydrolysis of other polymers, the ammonia and phosphates act as a buffering system and prevent fluctuations in pH, which could otherwise be detrimental to the tissue [61]. Although the exact mechanism of degradation is not known, there are several pathways that have been proposed (see Scheme 2) [37]. Overall, the first two steps of the degradation result in the hydrolysis of the ester of the amino acid, forming an alcohol, and detachment of the amino acid from the polyphosphazene backbone forming the amino acid itself. The backbone of the polyphosphazene is then hydrolyzed to phosphates and ammonia. The formation of phosphates during the degradation process was verified through the addition of silver nitrate or zirconyl chloride which forms a yellow silver phosphate or white zirconyl phosphate precipitate, respectively [37]. The amino acids and ammonia degradation products can be demonstrated by ninhydrin test, which detects ammonia and primary and secondary amines whereas as ^1^H NMR spectroscopy can be utilized for detecting alcohols.

Another important factor with regard to biodegradability is the rate at which the material degrades since this can limit potential applications. It is important when designing a scaffold that the material degrades at a rate that is similar to the rate of tissue growth or therapeutic release rate depending on the application. For tissue engineering, if the scaffold material degrades too quickly there will be insufficient support for the underdeveloped tissue and mechanical weakness will ensue. If the material degrades too slowly or not at all, it may need to be surgically removed which could in turn damage the neotissue and cause problems with mismatched mechanical properties relative to the natural tissue [62]. For therapeutic delivery, it is desirable to reduce burst release corresponding to rapid degradation and poor release corresponding to very slow degradation. In order to determine the degradation rates of poly(amino acid ester)phosphazenes, the influence of changing the types and ratios of side chain substituents on the degradation properties of the polymers is an important factor.  Table 1 provides an overview of the degradation studies that have been performed on polyphosphazenes substituted with amino acid esters and other cosubstituents.

In view of this, the degradation rates of poly(amino acid ester)phosphazenes with different amino acids and different esters of the amino acids were studied in solution- and solid-state degradation, although solid-state degradation is more representative of how degradation would occur with* in vivo* scaffold materials and is the method that will be discussed [37]. The effect of changing the ester group was investigated using glycine-based poly(amino acid ester)phosphazenes including poly[bis(methyl glycinat-N-yl)phosphazene] (PNMG), poly[bis(ethyl glycinat-N-yl)phosphazene] (PNEG), poly[bis(*tert*-butyl glycinat-N-yl)phosphazene] (PNtBG), and poly[bis(benzyl glycinat-N-yl)phosphazene] (PNBzG). In this systematic study, the molecular weight decline was in the order of PNBzG < PNtBG < PNEG < PNMG, with PNMG having the greatest decrease in molecular weight. This showed that as the hydrophobicity of the ester group increased (from methyl to benzyl), the molecular weight decline of the polymer decreased. The decreased molecular weight decline is due to the inability of water to approach the polymer due to its hydrophobicity, and therefore the hydrolysis of the material is limited. The effect of changing the amino acid using poly[bis(methyl glycinat-N-yl)phosphazene] (PNMG), poly[bis(methyl alaninat-N-yl)phosphazene] (PNMA), poly[bis(methyl valinat-N-yl)phosphazene] (PNMV), and poly[bis(methyl phenylalaninat-N-yl)phosphazene] (PNMF) showed that the molecular weight decline increased in the order of PNMF < PNMV < PNMA < PNMG. This trend was observed since the hydrophobicity of the polymer increased as larger nonpolar side chain amino acids, like phenylalanine, were incorporated into the polyphosphazene. This study was a good initial demonstration of the biodegradability and hydrolysis properties of different poly(amino acid ester)phosphazenes, although a more suitable degradation medium would be phosphate buffer solution (PBS) at 37°C, which is more representative of the body fluid pH, temperature, and ion concentrations.

The effect of the types of side groups on the degradation rates of L-alanine cosubstituted polyphosphazenes, specifically PNEA, poly[(ethyl alanato)_1_ (ethyl glycinato)_1_phosphazene] (PNEAEG), PNEAmPh, and PNEAPhPh, were also reported in a separate study [23]. As may be expected, the ethyl glycinato substituted phosphazene (PNEAEG) had the fastest molecular weight decline, whereas the biphenyl substituted phosphazene (PNEAPhPh) had the slowest molecular weight decline. The PNEAEG material hydrolyzed so quickly that molecular weight could not be evaluated beyond week two of the degradation study. It was noted that the pattern of molecular weight decline showed a quicker degradation rate for the smaller, more hydrophilic substituent polymers as compared to those substituted with large bulky hydrophobic substituents. Compared to imidazolyl side groups, increasing the amount of ethyl glycinato groups increased the degradation rate of the polymer, indicating that the incorporation of less sterically hindered, more hydrophilic groups causes the polymers to degrade more quickly [56]. The results of the study were successful in demonstrating the tunability of degradation properties of cosubstituted polyphosphazenes, which is a key requirement in the development of a biomaterial for tissue engineering applications. Overall, this study effectively showed the ability to tune degradation rates of poly(amino acid ester)phosphazenes through careful selection of side group substituents. One thing to consider when selecting side groups for biodegradable polyphosphazenes that incorporate amino acids is the degradation by natural enzymes found* in vivo*. If the enzymes are capable of recognizing the amino acid, enzymatic and hydrolytic degradation together may increase the degradation rate of the polymer as compared to hydrolysis alone. Also, if the enzymes in the native tissue are capable of recognizing the polyphosphazene-bound amino acids, their ability to interact with them may be sterically hindered if bulky substituents are cosubstituted on the polymer, causing further complications in approximating degradation rates of poly(amino acid ester)phosphazenes from* in vitro* studies.* In vivo* degradation studies showed substantial decline in molecular weight for the PNEA and PNEAmPh implants after 12 weeks, 80% and 98%, respectively [47]. PNEAPhPh, on the other hand, did not experience as great of a molecular weight decline as the other two implants and had a molecular weight decline of only 63% after 12 weeks. This is presumably due to the increased hydrophobicity of the biphenyl substituent, which limits the approach of water to the polymer backbone and therefore slows its hydrolysis. This study demonstrated the* in vivo *biodegradability of poly(amino acid ester)phosphazenes, as well as their biocompatibility. We should caution that the implant in this cited study was designed for bone tissue engineering applications and, as such, it is implanted into a region of the rat where bone tissue is the predominant tissue type. Naturally occurring enzymes, which have the potential to significantly influence degradation rates if they recognize the materials, have different abundance across different types of tissues. Therefore, if the poly(amino acid ester)phosphazenes investigated in this study are to be applied to other tissue engineering applications, their degradation rates in those tissues may vary dramatically from those presented here due to differences in enzymatic degradation. The relative abundance of water in a tissue also determines rates of hydrolysis and the materials could therefore show significantly different hydrolytic degradation rates in different tissues.

Other studies were conducted on the effects of changing ratios of substituents on the degradation rates of the polymers [41]. Mass loss measurements following PBS incubation focused on cosubstituted polyphosphazenes of ethyl 2-(O-glycyl) lactate and ethyl glycinato. Decreasing the ratio of ethyl glycinato : ethyl 2-(O-glycyl) lactate, for materials with varying side chain ratios between 100% ethyl glycinato: 0% ethyl 2-(O-glycyl) lactate and 75% ethyl glycinato: 25% ethyl 2-(O-glycyl) lactate, increased the mass loss rate of the polymer. This is due to the increased hydrolytic sensitivity of ethyl 2-(O-glycyl) lactate, in comparison to ethyl glycinato, which encourages polymer degradation and therefore mass loss. Even though mass loss is not a direct indication of molecular weight decline [23], it is still a good indicator of the* relative* degradation rates of the polymers and, as such, it can be approximated that polymers with a higher ratio of ethyl glycinato substitution degrade less quickly than those with increased levels of ethyl 2-(O-glycyl) lactate. This study was useful in demonstrating the effect that varying ratios of substituents with different hydrolysis-sensitivities and solvation properties has on the degradation rates of the polymers, which can be useful for tailoring degradation properties of cosubstituted polyphosphazenes according to their specific biomedical applications. Furthermore, the degradation properties of depsipeptide-substituted polyphosphazenes have also been studied [63–65]. Depsipeptides are short chain amino acid sequences that contain at least one ester linkage in place of an amide bond in the backbone of the peptide chain. Although research on these types of polymers will not be discussed in detail in this review, it is important to mention their role in developing suitable biomaterials for biomedical applications. The reason that these polymers have been included in this review is that they are a good preliminary model for poly(amino acid ester)phosphazenes that have been functionalized with bioactive molecules, which are typically proteins and short peptide chains. The depsipeptide-type bonding in these functionalized polymers comes from the amide linkages throughout the biomolecule and a potential ester linkage through the carboxylate functionality of an amino acid side chain (e.g., aspartic and glutamic acid). The incorporation of these biomolecules can significantly enhance cellular interactions and biomimetic properties of the materials, making them better candidates as biomaterials. Research on these polyphosphazene materials has shown their biodegradability, therefore suggesting their potential as biomaterials for tissue engineering and other biomedical engineering applications [41].

Taken together, these studies have been able to demonstrate not only that poly(amino acid ester)phosphazenes are biodegradable but also that their degradation rates can be tuned by changing a variety of factors, such as type and ratio of side groups. The fact that bioerodability studies have been performed* in vitro* in body fluid simulating solutions and* in vivo* in rat models suggests that these polyphosphazene materials are suitable for use in tissue engineering applications such as scaffold biomaterials as well as for other biomedical applications that require the use of degradable materials.

### 3.4. Mechanical Properties of Polyphosphazenes

**Figure 3:**
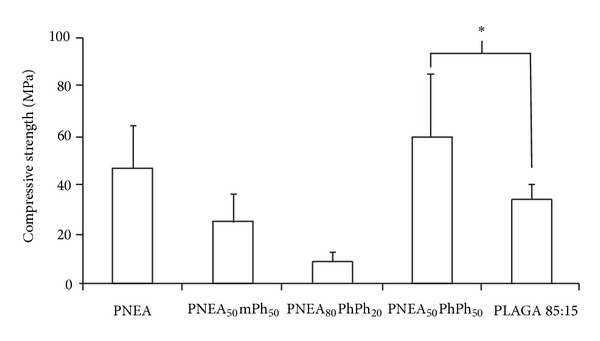
Compressive strengths of alanine-based amino acid ester phosphazenes in comparison to poly(lactic acid-co-glycolic acid) [PLAGA (85 : 15)]. The alanine-based polyphosphazenes presented are poly[bis(ethyl alanato)phosphazene] (PNEA), poly[(50% ethyl alanato) (50% methyl phenoxy)phosphazene] (PNEA_50_mPh_50_), poly[(80% ethyl alanato) (20% phenyl phenoxy)phosphazene] (PNEA_80_PhPh_20_), and poly[(50% ethyl alanato) (50% phenyl phenoxy)phosphazene] (PNEA_50_PhPh_50_). The ∗ indicates results that are significantly different (*P* < 0.05, *n* = 6). Reprinted from [70] with permission from Elsevier.

In order to produce clinically viable tissue-engineered products, the mechanical properties of the constructs must match the properties of the natural tissues. If the mechanical properties, such as compressive strength and tensile strength, are not comparable to those of native tissues, problems with mismatch arise which often lead to failure of the tissue-engineered construct [66–69]. There have been only a limited number of studies carried out to investigate the mechanical properties of poly(amino acid ester)phosphazene materials. One such study was conducted by Sethuraman et al. [70] who investigated the mechanical properties of alanine-based polyphosphazenes for their application as bone tissue engineering biomaterials (Figure 3). For these studies, polyphosphazenes were compared with the current standard for bone tissue engineering applications, PLAGA (85% lactic acid: 15% glycolic acid). Cylindrical discs of each polymer were initially subjected to a compressive force of 1500 pounds per square inch (psi) for 15 min and analyzed using a uniaxial compressive testing instrument set with the following parameters: 500N load cell and 10 mm/min compression rate, until material failure. The compressive strengths of PNEA and PNEAmPh were comparable to that of PLAGA (34.9 ± 5.7 MPa), with compressive strengths of 46.61 ± 17.56 MPa and 24.98 ± 11.26 MPa, respectively. PNEAPhPh on the other hand had a compressive strength that was significantly higher than that of PLAGA due to the large aromatic groups increasing steric bulk and decreasing torsion of the polymer backbone. Together, these increase the rigidity of the material and modulate its compressive properties. Therefore, it can be noted that the mechanical properties, like the degradation properties, of polyphosphazene materials can be tailored based on their proposed applications by changing the side group substituents. The tensile strength and elasticity of several L-alanine-based polyphosphazene materials (namely, PNEAEG, PNEA, PNEAmPh, and PNEAPhPh) were determined using microtensile testing techniques [70]. It was shown that increasing the steric bulk of the cosubstituent increased both the tensile strength and elasticity of the material with more of an impact being observed as the side chain is changed from a small amino acid such as glycine or alanine ethyl ester, such as in PNEAEG and PNEA, to large aromatic substituents, such as in PNEAmPh and PNEAPhPh. This is because introducing large aryloxy substituents affects the glass transition temperature and molecular weight of the polymer, which in turn affects the mechanical properties of the material. Overall, this study shows how the mechanical properties of a polyphosphazene material can be tailored simply through cosubstitution of large aromatic groups alongside amino acid esters.

Both of the above studies showed that changing the types and ratios of side group chemistries of polyphosphazene materials can modulate mechanical properties, such as compressive strength, tensile strength, and elasticity. Therefore, the mechanical properties of these materials can be tuned to suit the intended application, making polyphosphazenes useful as biomaterials in a wide range of different biomedical applications. One concern here is that changing the side groups affects not only mechanical properties but also degradation rates and therefore adapting the side chains to obtain suitable mechanical properties may cause the degradation rate of the material to be either too fast or too slow for the application, which is undesirable. As such, it is suggested that research into other methods to control mechanical properties that do not influence erosion properties be developed. For example, it may be useful to investigate the effects of different processing methods or scaffold preparation techniques (e.g., electrospinning versus solvent casting and particulate leaching) on mechanical properties.

## 4. Tissue Engineering and Drug Delivery Applications of Polyphosphazenes

Although many biomaterials have been previously investigated for tissue engineering applications, there have been limitations to each, such as acidic degradation products, as was discussed earlier. Therefore, once polyphosphazene materials were studied and proven suitable for biomedical applications according to their biocompatibility, biodegradability, and mechanical properties, they were implemented into tissue engineering research. The biomaterials are typically used to construct three-dimensional (3D), porous, biodegradable scaffolds, which temporarily support and direct tissue growth until the natural extracellular matrix (ECM) develops. A variety of polyphosphazenes have been investigated as scaffold materials for use in bone and skeletal tissue engineering [56, 71–74], nerve guides [60], and blood contact materials [75] (e.g., coatings for implants and blood dialysis devices).

### 4.1. Bone Tissue Engineering

The majority of research to date focused on polyphosphazenes as materials for bone and skeletal tissue engineering applications. Laurencin and coworkers have extensively studied these materials and their interactions with osteoblast type cells to determine their suitability for bone grafts and implants. For example, in 1996 [73], they developed 3D and 2D matrices of amino acid-based polyphosphazene, on which they seeded osteoblast cells. They observed that the pores of the 3D constructs resembled, in shape and size, those of natural bone tissue, specifically trabecular bone. They also noted that the 3D polyphosphazene scaffolds were able to promote osteoblast adhesion and proliferation throughout the entire 21-week period of their study, whereas adhesion to the 2D scaffolds was not as effective. Overall, this study gave a good indication that polyphosphazene materials were suitable for bone tissue engineering. Ambrosio et al. [71] also investigated the applicability of polyphosphazenes to bone tissue repair through the development of polyphosphazene-hydroxyapatite composites. They formed these composites (in a 1 : 3 ratio of polymer:ceramic) by dissolving the polymer in THF, mixing with hydroxyapatite particles, vortexing the mixture, and precipitating the mixture with hexanes to form a putty-like material from which cylindrical samples were formed. The composite material interacted favorably with MC3T3-E1 cells (osteoblast-like cell line) and demonstrated improved cell adhesion and proliferation in comparison to polystyrene-coated tissue culture plates (TCPS). They also showed that the composites had mechanical properties suitable to bone tissue engineering applications and that these properties were maintained throughout the degradation process of the material. This study demonstrated the utility of polyphosphazenes as biomaterials for bone tissue engineering purposes. More recently, Morozowich et al. [76] investigated the possibility of incorporating antioxidants into polyphosphazene materials to enhance their suitability as biomaterials for hard tissue engineering applications, such as bone tissue. They were capable of synthesizing ferulic acid, an antioxidant, and amino acid ester cosubstituted polyphosphazenes that showed degradation and UV-crosslinking properties suitable for hard tissue engineering applications. This suggested the material's potential use for these applications, although cytotoxicity has yet to be fully investigated. Although polyphosphazenes are thought to be osteoinductive materials because of their phosphorus-containing feature, they appeared to be less supportive to cell growth compared with the commonly used aliphatic polyesters. Muscle-inspired modification of fibrous polyphosphazene mats with poly(dopamine) is reported to overcome this apparent limitation [77].

### 4.2. Nerve Tissue Engineering

As alluded earlier, Langone et al. [60] used polyphosphazene materials towards nerve tissue engineering that investigated polymeric tubular nerve guides as prosthetics to promote nerve regeneration. They showed that poly[(ethyl alanato)_1.4_ (imidazolyl)_0.6_ phosphazene] tubular constructs were capable of promoting the* in vivo* reattachment of experimentally transected rat sciatic nerves and that the new tissue was populated with cells similar to native neural tissues. In a similar study [78], poly[bis(ethyl alanato)phosphazene] constructs were made, for neural tissue engineering, by dipping a glass capillary into a polymer solution and allowing the solvent to evaporate, leaving only the polymeric material. This process was repeated until a polymer film of appropriate thickness was formed, at which point the glass capillary and polymer coating were dried and finally the glass capillary was removed from inside the polyphosphazene construct. These constructs were then implanted* in vivo* into Wistar rats which had their right ischiatic nerve transected and the excised portion was replaced with the polymer conduit. The polyphosphazene constructs remained implanted for time periods of 30, 90, and 180 days and were compared to control experiments where the excised area of the right nerve was replaced with a portion of the left ischiatic nerve. Studies showed little to no toxicity of the absorbable polyphosphazene material, as well as nerve regeneration properties including myelinated and unmyelinated nerve fibers similar to the control autologous graft. Overall this work showed the successful use of polyphosphazene-based materials for the development of nerve guides in the regeneration of neural tissue.

More recently, Zhang et al. [79] have studied polyphosphazenes as conductive and degradable polymers for use in nerve tissue engineering. Conductivity is an important aspect of nerve tissue engineering considering that neural signals are propagated along nerve cells via electrical charges and therefore the polymers that are used to regenerate these tissues must be capable of transmitting waves of electricity. A cosubstituted polyphosphazene material consisting of parent aniline pentamer (PAP) and glycine ethyl ester (GEE) was synthesized and formed into thin films for degradation and biocompatibility testing. The poly[(glycine ethyl ester)(aniline pentamer) phosphazene] (PGAP) polymer was shown to have good electroactivity using cyclic voltammetry measurements meaning that the material would be suitable for propagating neural signals. Thin films of both PGAP and poly[bis(glycine ethyl ester)phosphazene] (PGEE) materials were subjected to degradation studies in PBS at 37°C over a study period of 70 days and showed mass losses of about 50% and 70%, respectively. The mass loss of the PGAP was less than that of the PGEE due to the increased hydrophobicity of the aniline pentamer side chain and therefore decreased rate of hydrolysis since the hydrophobic side chains sterically hinder the approach of water towards the backbone. Cell viability of the PGAP material was assessed using RSC96 Schwann cells and was compared to a thin film of poly-DL-lactic acid (PDLLA) as a control since it has extensively been shown to be biocompatible with numerous types of cells. RSC96 Schwann cells were chosen, as Schwann cells are integral parts in the peripheral nervous system not only as supportive cells but also to help with the myelination of the axons that propagate neural signals. The studies showed improved cell adhesion to the PGAP material in comparison to PDLLA, as well as no significant difference in cytotoxicity between the two materials. Overall, this work proves the usefulness of polyphosphazene-based materials as conductive and biodegradable polymers for nerve tissue engineering applications.

### 4.3. Tendon and Ligament Tissue Engineering

In 2012, Peach et al. [80] analyzed polyphosphazene-functionalized poly(*ε*-caprolactone) (PCL) materials for their application in tendon tissue engineering. In their studies, electrospun fibrous mats of PCL with average fiber diameters of 3000 ± 1700 nm coated with poly[(ethyl alanato)_1_ (p-methyl phenoxy)_1_phosphazene] (PNEA-mPh) were used to investigate cell behavior in response to the materials. Human mesenchymal stem cells (hMSC) were tested for their adhesion, infiltration, proliferation, and differentiation properties when exposed to the PNEA-mPh coated PCL constructs. The PNEA-mPh coated materials showed enhanced cell adhesion and infiltration as compared to the uncoated PCL fibers due to the increased surface roughness created by the dip-coating process. Cell proliferation was analyzed using the PicoGreen assay and showed that both materials were capable of sustaining long-term growth of the hMSCs* in vitro*. In order for tissue engineering constructs to be clinically relevant, oftentimes the cells that comprise the tissue must be differentiated to the appropriate phenotype; otherwise, the tissue may fail* in vivo*. In the case of tissue-engineered tendon grafts, the cells should undergo tenogenic differentiation by increasing tenomodulin expression, a late tendon differentiation marker protein. Both the uncoated and coated PCL fibrous mats expressed scleraxis equally, an early tendon differentiation marker protein, but the polyphosphazene coated mats showed increased tenomodulin expression indicating that this material was more phenotypically mature and a better candidate as a tendon regeneration material than the uncoated counterpart. The PNEA-mPh functionalized material also showed an increased ratio of collagen I to collagen III, as per real-time polymerase chain reaction (RT-PCR) analysis, which also indicates the maturity of the differentiated cells into tendon cells. Overall, this study was able to show the* in vitro* biocompatibility of polyphosphazene-coated materials towards human mesenchymal stem cells as well as their ability to modulate appropriately the cells' differentiation towards mature tendon cells.

Polyphosphazene materials with improved elastomeric properties were studied by Nichol et al. [81] in 2013 for their application in tendon and ligament tissue engineering applications. For this study, they investigated the influence of changing alkyl ester chain lengths between five and eight carbons on mechanical properties and degradation rates of L-alanine and L-phenylalanine alkyl ester polyphosphazene materials. They determined that the glass transition temperatures (*T*_*g*_) of the materials decreased with increasing alkyl ester chain length due to increased flexibility of the alkyl side chain and improved elastomeric properties of the polymer. It was also observed that the* T*_*g*_'s of the phenylalanine materials were higher than those of the alanine counterparts which is likely due to the increased bulkiness and steric hindrance of the aromatic side chain that in turn increases the rigidity of the overall polymer. For degradation studies, square (5 cm × 5 cm) solution-casted films were cut into 10 mg samples and placed in deionized water at pH 6.3 and 37°C for a time period of 12 weeks. After the specified weeks, the aqueous media was tested for pH and the remaining polymer sample was weighed and a GPC analysis was performed to determine mass loss and molecular weight decline, respectively. The resulting pH of the aqueous media varied between 5.2 and 6.8. Overall, the phenylalanine-based materials showed decreased molecular weight decline in comparison to the alanine-based materials independent of the length of the alkyl ester side chain. This is most likely due to the increased steric hindrance of the backbone due to the large aromatic rings in the side chain of phenylalanine which prevents water from reaching the bonds that are to be hydrolyzed. The phenylalanine materials were also capable of forming better films which makes them less susceptible to hydrolysis. Taken as a whole, the phenylalanine polyphosphazenes were shown to be the most suitable materials as scaffolds for soft tissue engineering applications due to their improved elastomeric properties and slow degradation rates.

### 4.4. Polyphosphazenes for Drug Delivery

**Figure 4:**
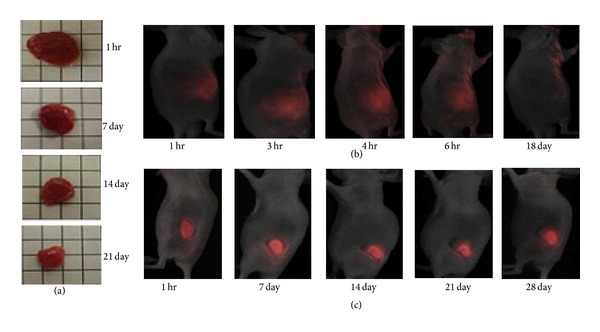
*In vivo* degradation process and* in vivo* localization of poly(organophosphazene)-doxorubicin (DOX) conjugate. Biodegradation as a time-dependent mass loss of intratumorally injected polymer-DOX conjugate (a). Time-dependent fluorescence image of intratumorally injected DOX solution (30 mg/kg) (b). Time-dependent fluorescence image of intratumorally injected poly(organophosphazene)-DOX conjugate hydrogel (100 mL, equivalent to 22.3 mg/kg of DOX) (c). Reprinted from [51] with permission from Elsevier.

Poly(organophosphazene)s were tested as delivery vehicles for the anticancer drug doxorubicin (DOX) [51]. A polyphosphazene with L-isoleucine ethyl ester (IleOEt), glycine glycine allyl ester (GlyGlyOAll), and *α*-amino-*ω*-methoxy-poly(ethylene glycol) (AMPEG 550) substituents was synthesized and subsequently conjugated with DOX through the pendant carboxylic acid groups after removing the allyl protecting groups on glycine glycine (poly[(IleOEt)_1.22_ (GlyGlyOH)_0.07_ (GlyGlyODOX)_0.05_ (AMPEG550)_0.66_ phosphazene]). These materials were shown to be injectable as a solution and precipitate into a gel material upon heating which is suitable for targeted drug delivery applications as it maintains the drug in the desired location, especially tumor sites. The material was tested* in vitro* for degradation properties, drug (DOX) release profile, and antitumor activity. Degradation studies and release profiles were performed in PBS (0.01 M, pH 7.4) at 37°C over 30 days. The mass loss after 30 days was approximately 60% and the molecular weight decline was slightly less than 40%. The DOX release profile demonstrated a sustained release of the drug which is ideal for most drug delivery techniques. The* in vitro *antitumor activity of the DOX-conjugated polyphosphazene material was compared to both the polyphosphazene material alone and DOX alone, as controls, using human breast cancer (MCF-7) and cervical carcinoma (HeLa) cell lines. It was shown that the unconjugated polyphosphazene did not act as an antitumor agent with either cell type with an inhibitory concentration (IC_50_) greater than 30 *μ*M. The DOX-conjugated polyphosphazene on the other hand showed IC_50_ similar to those of the DOX alone for both MCF-7 and HeLa cell lines with approximately 1 *μ*M and 0.2 *μ*M, respectively.* In vivo* antitumor activity analyses were performed on mice models that had been subcutaneously implanted with tumor cells (SNU-601 human gastric cancer cell line). The mice were injected with two concentrations of the DOX-conjugated polyphosphazene, a solution of free DOX, and saline as a control. The tumor volume of the saline control steadily increased throughout the 28-day span of the study, whereas the tumor volumes decreased for all of the DOX containing solutions indicating growth inhibition of the tumor (Figure 4). The free DOX solution showed tumor suppression of about 62% by day 4, followed by a slight increase in relative tumor volume at day 6, and death of animal by day 12 due to the toxicity of high levels of DOX. The polyphosphazene-DOX conjugates on the other hand showed prolonged tumor suppression throughout the entire study period. The higher dosage of DOX-conjugated (44.5 mg of DOX per kg weight of mouse) material showed tumor suppression of 47%, 55%, and 75% at 4, 12, and 28 days and was not so toxic as to kill the animal model, unlike the free DOX solution. This study shows the great potential of polyphosphazene materials, even over traditional methods such as bolus injections, for sustained drug delivery and other biomedical applications that require gradually degrading biomaterials.

Recently, Song and coworkers have been developing poly(organophosphazene)s that are injectable and contain anticancer agents such as silibinin [53] and camptothecin [82]. They used L-isoleucine ethyl ester and deprotected glycine glycine allyl ester substituents and were able to conjugate the drugs through the pendant carboxylic acid groups. Both studies investigated the* in vitro* degradation properties and drug release profiles of the two conjugated materials. In both cases the drug showed sustained release over the time frame of the study which is especially beneficial for drugs that may be lethal at high concentrations and cannot, therefore, be administered as bolus injections.* In vitro *and* in vivo* studies of antitumor activity were performed on both the silibinin- and camptothecin-conjugated polyphosphazene materials and both proved to have tumor inhibition effects against HT-29 colon cancer cell line. For the* in vivo* analyses, solutions of both the polymer-drug conjugate and drug only were injected into a site previously implanted with an HT-29 cell xenograft and in all cases the polymer-drug conjugates were just as effective at tumor inhibition as the drug alone but without the toxic side effects of the drug only solution. In the silibinin-based study the researchers also performed Western blot analyses and determined that silibinin elicited an antiangiogenic effect as observed by the protein compliment being expressed by the cells. Overall, poly(organophosphazene)s conjugated with anticancer agents have shown to be successful as injectable thermosensitive hydrogels for targeted drug delivery.

## 5. Polyphosphazene Blends as Biomaterials

Despite polyphosphazenes having inherent tunability through their side chains, occasionally this is insufficient to match the required material properties of specific biomedical applications and thus polyphosphazene blends have also been explored as potential biomaterials. Lin et al. [52] investigated the effect of varying polymer ratios on the morphology of electrospun mats of poly[(alanine ethyl ester)_0.67_ (glycine ethyl ester)_0.33_ phosphazene] (PAGP) and gelatin. The polymer ratios tested were from 0 to 90 weight percent (wt%) gelatin to PAGP and these resulted in mean fiber diameters between 300 nm and 1 *μ*m. Higher gelatin content led to homogeneously distributed fibers with larger diameter fibers. At lower gelatin ratios (below 50 wt%), fibers showed a heterogeneous morphology with a gelatin core and PAGP shell. Also, the water contact angles of the materials showed that the PAGP material is significantly more hydrophobic than the gelatin and the overall surface hydrophobicity of the material can be tailored by adjusting the ratios of the two copolymers in the blend. This tunability of surface hydrophobicity and fiber diameter by varying ratios of the copolymers in polyphosphazene blends may further increase their utility in biomedical applications in the future.

Blends of polyphosphazenes and polyesters as biomimetic scaffolds for bone regeneration have also been studied [83–85]. For instance, nanofibers of PLAGA, poly[(glycylglycine ethyl ester)_1_ (phenyl phenoxy)_1_ phosphazene] (PPHOS), and blends of the two together were formed via electrospinning techniques [83]. The glycine dipeptide was incorporated to minimize phase separation of the two polymers in the blend fibers by hydrogen bonding with PLAGA. The large aromatic phenyl phenoxy groups were used to maintain the mechanical properties, such as compression resistance, and hydrophobicity of the blend materials. Nonwoven mats with fiber diameters between 50 and 500 nm had similar elastic modulus and ultimate tensile strength to PLAGA indicating their appropriate mechanical properties for bone tissue engineering applications.* In vitro*, these 3D biomimetic scaffolds were capable of promoting cell infiltration, as indicated by the migration of cells from the blend layers to the interlayer space, and extracellular matrix deposition by the osteoblast cells, as shown by the phenotype marker expression including ECM proteins such as osteopontin. Overall, this study shows the success of polyphosphazene blend materials as potential biomaterials for biomedical applications such as bone tissue engineering.

## 6. Conclusions and Future Outlook

Throughout this review paper, the potential of polyphosphazenes for use in biomedical applications has been explored. Rather than focusing on the applications alone, this review attempted to provide a larger overview of synthesis techniques and in-depth rationale of polyphosphazene polymers as biomaterials, specifically their biocompatibility, biodegradability, and mechanical properties, all of which are key characteristics of biomaterials. Polyphosphazenes are currently being extensively studied as scaffold materials and drug delivery devices, although their utility in other biomedical applications have not yet been fully investigated. As interest in the area of biocompatible poly(organo)phosphazenes grows, it is expected that these materials will be employed for other tissue engineering applications, such as tendon and blood vessel engineering, as well as a wide range of other biomedical applications.

## Figures and Tables

**Table 1:** Summary of *in vitro* degradation studies of poly(amino acid ester)phosphazenes and their cosubstituted polyphosphazenes. The ester refers to the chain attached to the carboxyl terminus of the amino acid. The detailed degradation profiles can be found in the cited papers.

Amino acid	Ester	Cosubstituents	Study length	Reference(s)
Glycine	Methyl		35 days	[37]
	Ethyl		35–120 days	[22, 37, 38]
		Alanine ethyl ester (50%)	7 weeks	[23]
		p-Methyl phenoxy (25–90%)	7 weeks	[56]
		Ethyl-2-(O-glycyl)lactate (0–25%) and phenylalanine ethyl ester (70%)	120 days	[22]
	*t*-Butyl		5 weeks	[37]
	Benzyl		5 weeks	[37]
Alanine	Methyl		5 weeks	[37]
	Ethyl		7 weeks	[23, 38]
		p-Methyl phenoxy (50%)	7 weeks	[23]
		p-Phenyl phenoxy (50%)	7 weeks	[23]
	Benzyl		7 weeks	[38]
Valine	Methyl		5 weeks	[37]
Phenylalanine	Ethyl		5 weeks	[37]
